# Serum neutrophil gelatinase-associated lipocalin and resistin are associated with dengue infection in adults

**DOI:** 10.1186/s12879-016-1759-9

**Published:** 2016-08-22

**Authors:** Kuan-Ting Liu, Yao-Hua Liu, Chun-Yu Lin, Ming-Ju Tsai, Ya-Ling Hsu, Meng-Chi Yen, Po-Lin Kuo

**Affiliations:** 1 Graduate Institute of Clinical Medicine, College of Medicine, Kaohsiung Medical University, No.100, Shih-Chuan 1st Road, Kaohsiung, 807 Taiwan; 2 Department of Emergency Medicine, Kaohsiung Medical University Hospital, Kaohsiung Medical University, No.100, Tzyou 1st Road, Kaohsiung, 807 Taiwan; 3 School of Medicine, College of Medicine, Kaohsiung Medical University, Kaohsiung, 807 Taiwan; 4 Division of Infectious Diseases, Department of Internal Medicine, Kaohsiung Medical University Hospital, Kaohsiung Medical University, Kaohsiung, 807 Taiwan; 5 Division of Pulmonary and Critical Care Medicine, Kaohsiung Medical University Hospital, Kaohsiung Medical University, Kaohsiung, 807 Taiwan; 6 Graduate Institute of Medicine, College of Medicine, Kaohsiung Medical University, Kaohsiung, 807 Taiwan; 7 Institute of Medical Science and Technology, National Sun Yat-Sen University, Kaohsiung, 804 Taiwan

**Keywords:** Dengue, Neutrophil gelatinase-associated lipocalin (NGAL), Resistin, Serum

## Abstract

**Background:**

Dengue is a major health problem in tropical areas, including Taiwan. Dengue virus infection affects various types of cells and results in elevation of serum inflammatory molecules. Because these molecules may be associated with dengue virus infection, the aim of this study was to identify novel molecules in febrile patients with dengue infection. In addition, we determined whether these molecules were correlated with the count of leukocytes and platelets.

**Methods:**

Febrile adults (Age >18 years old) who presented to the emergency department and were confirmed dengue virus infection were enrolled in this study. Serum from dengue patients and healthy controls was collected and serum level of sepsis-associated inflammatory molecules was measured by Luminex assay.

**Results:**

Elevated level of macrophage migration inhibitory factor, soluble vascular cell adhesion molecule-1, sFasL, resistin and interferon-γ were detected in patients’ serum. Higher levels of neutrophil gelatinase-associated lipocalin (NGAL) and resistin were detected in dengue patients with normal leukocyte count and all dengue patients, respectively. Furthermore, the serum level of NGAL, but not resistin, was correlated with cell count in dengue patients.

**Conclusion:**

Our results revealed that resistin and NGAL are novel dengue-associated molecules. These results may help elucidate the regulatory mechanisms of anti-dengue immune responses.

## Background

Dengue is a mosquito-borne viral disease caused by four serotypes of dengue virus and is endemic in tropical and subtropical areas including Southeast Asia [1]. A report from World Health Organization indicated the incidence of dengue fever has risen 30-fold in the past 50 years [2]. A recent study revealed that 390 million people were estimated to be infected with dengue per year [3]. According to the Taiwan National Infectious Disease Statistics System, more than 20,000 cases of dengue fever were diagnosed in southern Taiwan, including Kaohsiung city and Tainan city, since 2014 [4]. It is therefore a serious threat to public health in Taiwan and tropical countries.

Infection with any dengue serotype causes a wide spectrum of symptoms, ranging from a mild flu-like syndrome (dengue fever) to a severe syndrome (dengue hemorrhagic fever and dengue shock syndrome) [2]. Onset of fever is observed in dengue patients during the acute febrile phase of dengue. The acute phase of illness lasts for 3–7 days, and then the convalescent phase lasts for several days to several weeks [5, 6]. Because dengue virus infects various cell types, including peripheral leukocytes and endothelial cells [7], the levels of multiple cytokines and chemokines such as macrophage migration inhibitory factor (MIF), tumor necrosis factor alpha (TNF-α), interleukin (IL)-1β, and interferon-γ (IFN-γ), increase at different time points during the disease course [8–10]. The serum level of neutrophils-secreted elastase in patients with dengue shock syndrome is higher than in dengue patients without shock [11, 12]. In addition, dengue virus-infected patients have elevated levels of soluble intercellular cell adhesion molecule 1 (sICAM-1) and soluble vascular adhesion molecule-1 (sVCAM-1), which may regulate the activation and damage of endothelial cells [13]. Fas/Fas ligand (FasL) pathways participate in dengue virus-induced apoptosis of endothelial cells [14]. On the other hand, low platelet count and low leukocyte count are often observed in dengue patients [15, 16]. Platelets-secreted molecules may play a role in both dengue pathogenesis and inflammation regulation because dengue virus also alters platelets-leukocytes and platelets-endothelial cells interactions [17]. Activated platelets affect the production of IL-10 and TNF-α from mononuclear cells [18]. Because some of the aforementioned molecules were common markers in both sepsis and dengue [19], we were interested in whether other sepsis-associated molecules were also associated with dengue infection.

This study aimed to determine novel dengue-associated molecules in febrile patients. The Luminex assays were used for analyzing the sepsis-associated molecules in serum of dengue patients and healthy controls. In order to determine the potential association between these molecules and cell types (such as leukocytes and endothelial cells), the expression pattern of serum molecules in online available dataset was analyzed, either. Because low white blood cell count and low platelet count are clinical features of dengue, we also investigated whether the concentration of these molecules were correlated with white blood cell count and platelet count.

## Methods

### Sample collection

Adult patients (age >18 years old) who presented to the emergency department of Kaohsiung Medical University Hospital with fever (tympanic temperature >38.3 °C) from Sep 2014 to Dec 2014 were eligible for the study. After obtaining informed consent, 10 ml of blood was drawn and then the serum separated and stored in aliquots in −80 °C. Because dengue is a notifiable disease in Taiwan, serum should be collected from all patients with suspected dengue infection and sent to the Taiwan Centers for Disease Control (CDC), where final confirmation of the diagnosis was made (The commercial DENV Ag NS1 Strip [Bio-Rad] was used). Patients with dengue confirmed by CDC were selected and their sera were used for the following Luminex assay. The sera from healthy controls were also collected after obtaining informed consent.

### Quantification of sepsis-associated molecules

The concentration of serum molecules was determined by using Luminex technology. MILLIPLEX MAP Human Sepsis Magnetic Bead Panel 1 (macrophage migration inhibitory factor [MIF], soluble intercellular adhesion molecule 1 [sICAM-1], soluble Fas [sFas], soluble Fas ligand [sFasL], soluble vascular cell adhesion molecule 1 [sVCAM-1], and total plasminogen activator inhibitor-I [tPAI-I]) (Millipore, Billerica, MA, USA), MILLIPLEX MAP Human Sepsis Magnetic Bead Panel 3 (lactoferrin, elastase 2, neutrophil gelatinase–associated lipocalin [NGAL], resistin, and thrombospondin-1) (Millipore, Billerica, MA, USA) and Magnetic Luminex Performance Assay (Human High Sensitivity Cytokine Base Kit A, interferon-γ [IFN-γ]) (R&D Systems, Minneapolis, MN, USA) were used for determining the serum levels of these molecules according to the manufacturer’s instructions. Data was acquired on Luminex xMAP technology (Millipore, St Charles, MO, US). For concentration calculation, the calibration curve for each serum molecule was analyzed with a five parameter logistic curve fit curve through the Milliplex Analyst Software (Viagene Tech, Carlisle, MA, USA).

### Collection of independent microarrays dataset regarding dengue virus infection

The publicly available microarray dataset was obtained from NCBI Gene Expression Omnibus datasets (GEO dataset, http://www.ncbi.nlm.nih.gov/gds/) [20]. Briefly, we searched “dengue” at GEO datasets and then the results were filtered by the criteria “human” and “expression profiling by array.” After excluding the datasets from cell lines and peripheral blood mononuclear cells (PBMC) and datasets without healthy control, the dataset with GEO Series accession number of GSE51808 (http://www.ncbi.nlm.nih.gov/geo/query/acc.cgi?acc=gse51808) was chosen. The dataset contains healthy controls (9 healthy controls) and dengue patients (18 dengue fever patients and 10 dengue hemorrhagic fever patients) [21]. Expression values were adapted from GEO2R.

### Statistical analysis

Differences between two independent groups were analyzed with student’s T test (Two tailed). Comparisons between three groups were done with one-way ANOVA test, followed by Bonferroni’s multiple comparison test. The results were expressed as the median and the inter quartile range. Correlation was estimated with Spearman’s correlation. Graphs and statistical analysis were carried out using GraphPad Prism version 5.03 (GraphPad Software, San Diego, CA). *P*-value < 0.05 was considered significant.

## Results

### Identification of dengue-associated molecules in serum of dengue patients and healthy controls

Two hundred and seventy-four patients with non-severe dengue (145 males, median age is 42.5), and 33 healthy controls (20 males, median age is 37) were collected. Serum levels of MIF, sVCAM-1, sFasL, resistin and IFN-γ in dengue patients were significantly higher than those in healthy controls (Table 1). To the best of our knowledge, this is the first report describing the association between resistin and dengue infection. In order to compare the expression pattern of these molecules in patients’ serum with the expression pattern of these molecules in whole blood cells, we obtained microarray data from Gene Expression Omnibus (GEO, accession number: GSE51808, gene expression profile in whole blood cells) [21]. As shown in Table 2, elevated mRNA levels of MIF and decreased mRNA level of thrombospondin-1 were observed in dengue patients (including dengue fever and dengue hemorrhagic fever). Different expression pattern was observed in serum of dengue patients and RNA expression of whole blood cells. In whole blood cells, there was no difference was found in resistin level in dengue patients compared with that in healthy control (Table 2).

**Table 1:** Serum concentrations of sepsis-associated molecules in healthy donors and dengue patients

	Healthy controls (*n* = 33)	Dengue patients (*n* = 274)	*P* value
MIF	190.1 ± 27.83	753.6 ± 27.83	0.0046
sICAM-1	151,276 ± 12,035	381,200 ± 44,284	0.0776
sVCAM-1	487,114 ± 27,041	1,681,017 ± 30,861	<0.0001
sFasL	32.04 ± 1.657	76.04 ± 2.224	<0.0001
sFas	5428 ± 2622	5205 ± 325	0.8639
tPAI-1	117,506 ± 6263	122,256 ± 2207	0.4803
Elastase 2	76,746 ± 9427	73,292 ± 3601	0.7511
Lactoferrin	199,422 ± 22,270	244,062 ± 12,547	0.2263
NGAL	183,962 ± 16,769	272,870 ± 18,830	0.1047
Resistin	8778 ± 1099	17,451 ± 707.6	<0.0001
Thrombospondin-1	9,873,050 ± 214,185	9,749,456 ± 695,838	0.8513
IFN-γ	0.182 ± 0.003	5.377 ± 1.147	<0.0001

Note: Serum concentrations (pg/mL) of molecules were expressed in Mean ± SEM

**Table 2:** Gene expression between health donors and dengue patients (adapted from GEO dataset: GSE51808)

Gene symbol	Probe	Healthy (*n* = 9)	Dengue (*n* = 28)	*P* value
MIF	217871_PM_s_at	10.28 ± 0.05	11.29 ± 0.12	<0.0001
ICAM-1	202637_PM_s_at	7.34 ± 0.12	7.84 ± 0.16	0.0987
VCAM-1	203868_PM_s_at	3.39 ± 0.07	3.29 ± 0.08	0.4704
FasL	210865_PM_at	6.34 ± 0.30	6.07 ± 0.16	0.4339
Fas	216252_PM_x_at	8.51 ± 0.14	7.85 ± 0.19	0.0669
PAI-1	1568765_PM_at	4.92 ± 0.17	4.88 ± 0.09	0.6271
Elastase 2	206871_PM_at	4.65 ± 0.27	4.80 ± 0.30	0.7888
Lactoferrin	202018_PM_s_at	8.50 ± 0.42	9.43 ± 0.35	0.1748
NGAL	212531_PM_at	8.15 ± 0.18	8.76 ± 0.19	0.0975
Resistin	220570_PM_at	6.63 ± 0.38	7.01 ± 0.25	0.4565
Thrombospondin-1	215775_PM_at	3.90 ± 0.07	3.74 ± 0.05	0.0272
IFN-γ	210354_PM_at	5.80 ± 0.23	6.29 ± 0.16	0.1333

Note

The number in parentheses is the number of patients

The Dengue group includes patients with dengue fever (*n* = 18) and dengue hemorrhagic fever (*n* = 10)

Expression values of molecules were expressed in Mean ± SEM

### Sepsis-associated molecules in patients with leukopenia and thrombocytopenia

We further investigated whether the concentration of these molecules were associated with blood cell counts in dengue patients since leukopenia (<4000/mm^3^) and thrombocytopenia (<100,000/mm^3^) are common clinical features of dengue. The levels of sVCAM-1 and IFN-γ were significantly higher in both leukopenic and thrombocytopenic dengue patients than in other dengue patients, whereas the levels of elastase 2, lactoferrin, NGAL, and thrombospondin-1 were significantly lower in both leukopenic and thrombocytopenic dengue patients than those in other dengue patients (Tables 3 and 4). The result implies that NGAL is a novel molecule which might associate with anti-dengue immune responses. Similar expression pattern of these molecules was observed in the leukopenic group and thrombocytopenic group. The results might suggest the level of elastase 2, lactoferrin, NGAL, and thrombospondin-1 associate with the number of leukocytes and platelets.

**Table 3:** Serum concentrations of sepsis-associated molecules in dengue patients with different leukocyte counts

	Leukocyte count <4000/mm^3^ (*n* = 158)	Leukocyte count >4000/mm^3^ (*n* = 116)	*P* value
MIF	741.5 ± 86.56	770.1 ± 106.9	0.8338
sICAM-1	390,856 ± 63,423	368,049 ± 59,280	0.7996
sVCAM-1	1,861,371 ± 46,636	1,435,361 ± 34,827	<0.0001
sFasL	75.38 ± 2.79	76.94 ± 3.63	0.7295
sFas	5744 ± 502.9	4471 ± 337.5	0.0526
tPAI-1	120,103 ± 2986	125,189 ± 3258	0.2557
Elastase 2	60,422 ± 3308	90,821 ± 6909	<0.0001
Lactoferrin	200,423 ± 20,354	303,259 ± 21,099	<0.0001
NGAL	221,109 ± 8826	343,372 ± 42,051	0.0012
Resistin	17,016 ± 835	17,920 ± 1227	0.5706
Thrombospondin-1	8,699,875 ± 256,414	11,230,123 ± 321,044	<0.0001
IFN-γ	7.696 ± 1.91	2.219 ± 0.64	0.0180

Note: Serum concentrations (pg/mL) of molecules were expressed in Mean ± SEM

**Table 4:** Serum concentrations of sepsis-associated molecules in dengue patients with different platelet counts

	Platelet count <100,000/mm^3^ (*n* = 83)	Platelet count >100,000/mm^3^ (*n* = 191)	*P* value
MIF	816.7 ± 133.5	726.1 ± 77.27	0.5370
sICAM-1	463,596 ± 114,059	345,395 ± 39,751	0.2206
sVCAM-1	1,920,550 ± 45,670	1,576,926 ± 37,198	<0.0001
sFasL	73.75 ± 3.96	77.04 ± 2.76	0.4976
sFas	6274 ± 879	4741 ± 262	0.0299
tPAI-1	118,768 ± 4450	123,770 ± 2508	0.2984
Elastase 2	54,986 ± 4241	81,246 ± 4718	0.0007
Lactoferrin	173,218 ± 18,650	274,264 ± 15,551	0.0002
NGAL	206,081 ± 10,558	301,894 ± 26,368	0.0191
Resistin	18,640 ± 1213	16,934 ± 866	0.2687
Thrombospondin-1	7,557,301 ± 357,474	10,722,449 ± 232,877	<0.0001
IFN-γ	10.23 ± 3.45	3.26 ± 0.63	0.0051

Note: Serum concentrations (pg/mL) of molecules were expressed in Mean ± SEM

### Correlation between those molecules with leukocyte and platelet count in dengue patients

**Fig. 1:**
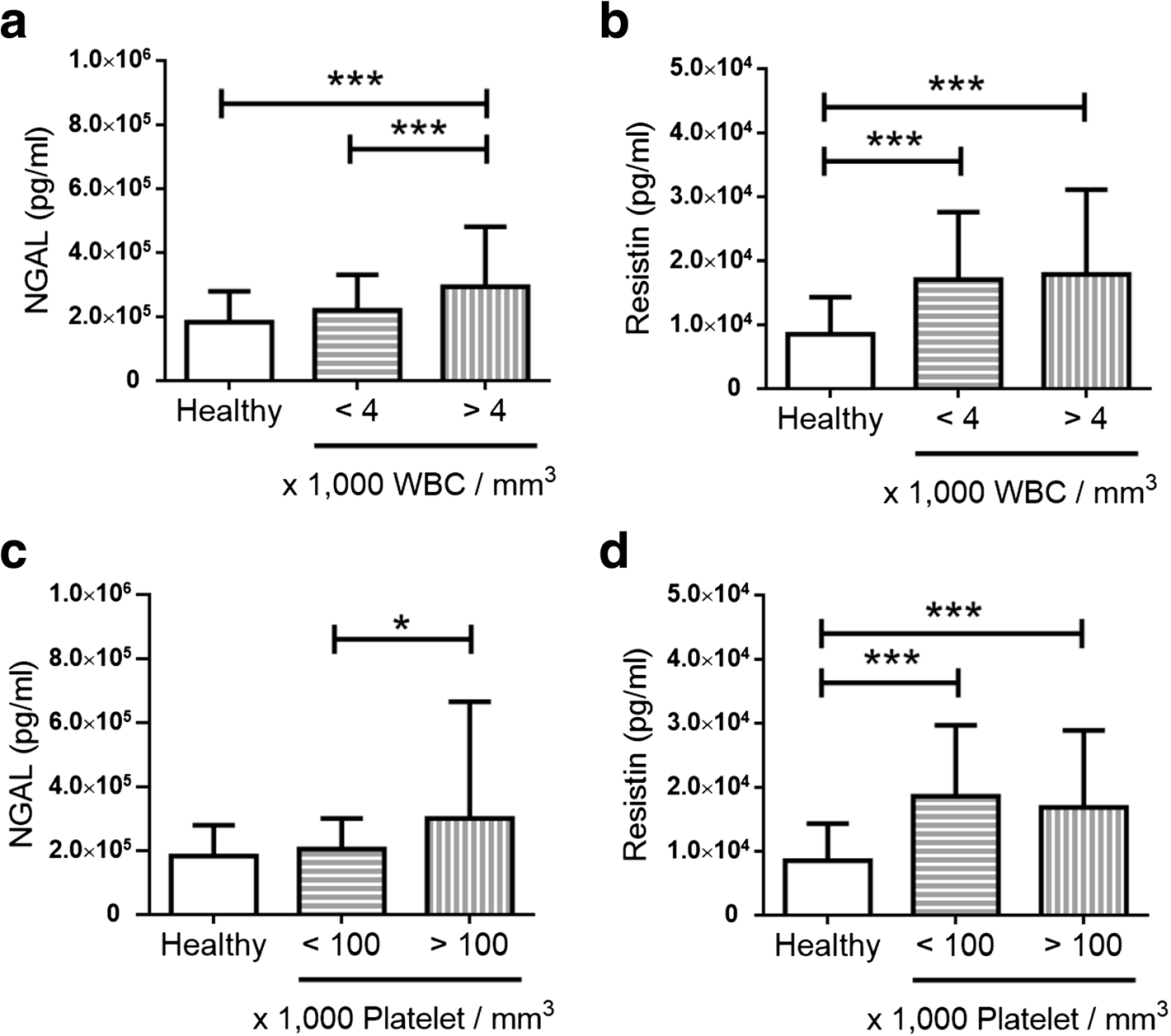
The levels of NGAL and resistin in healthy controls and dengue patients with different cell count. *Bar graphs* showing the serum concentration of (**a**) NGAL and (**b**) resistin in healthy controls and dengue patients with different leukocyte count; (**c**) NGAL and (**d**) resistin in healthy controls and dengue patients with different platelet count. All quantitative data are expressed as means ± SD. * *p* < 0.05; *** *p* < 0.0001

Increased serum level of resistin was observed in dengue patients. Serum NGAL level was significantly higher in dengue patients without leukopenia or thrombocytopenia than those with leukopenia or thrombocytopenia (Fig. 1). In contrast, serum resistin level was similar in the groups of dengue patients classified by the presence of leukopenia or thrombocytopenia (Fig. 1). The results further suggest the NGAL level correlates with the number of blood cells. Further analyses showed that serum level of NGAL, but not resistin, was correlated with leukocyte and platelet count in dengue patients (Table 5). Elastase 2 and lactoferrin were correlated with leukocyte and platelet count, either.

**Table 5:** Correlation between serum molecules and the blood cell counts in dengue patients

Cell count	Molecules	Spearman’s *R* value	*P* value
Leukocyte count	Elastase 2	0.2758	<0.0001
Leukocyte count	Lactoferrin	0.3508	<0.0001
Leukocyte count	NGAL	0.2764	<0.0001
Leukocyte count	Resistin	−0.0398	0.5120
Platelet count	Elastase 2	0.2774	<0.0001
Platelet count	Lactoferrin	0.3527	<0.0001
Platelet count	NGAL	0.2764	<0.0001
Platelet count	Resistin	−0.0420	0.4889

## Discussion

In 2014, there was a dengue fever outbreak in Taiwan and 96 % of patients were from Kaohsiung city [22]. A recent report indicates dengue virus 1 is dominant serotype [23]. Because patients were enrolled from Sep 2014 to Dec 2014 in Kaohsiung city, we supposed serotype I was predominant serotype in our study. Furthermore, a report indicated around 70 % of non-dengue virus 2 infection (dengue virus 1 and dengue virus 3) is primary infection in Taiwan [24]. It might imply that enrolled patients with primary infection were dominant. Our results showed the serum concentrations of MIF, sVCAM-1, sFasL, resistin, and IFN-γ were significantly higher in dengue patients than that in healthy controls. Previous studies have indicated that elevated serum levels of MIF, sVCAM-1, sFas, sFasL, and IFN-γ are associated with dengue [9, 10, 13, 14]. Similar expression pattern was observed in the present study. To the best of our knowledge, this is the first report describing elevated serum levels of resistin in dengue patients.

Previous studies have shown that elastase 2, lactoferrin, and NGAL are mainly secreted from neutrophils [25]. Elastase 2 and lactoferrin locate in azurophilic granules and specific granules, respectively [26]. Increasing serum levels of elastase 2 and lactoferrin are correlated with neutrophil degranulation and IL-8 is one of the important regulators for neutrophil degranulation in dengue-infected children [11, 27]. NGAL is a kind of protein inhibiting bacterial growth [28]. The role of NGAL is unclear in dengue infection. The plasma and urine NGAL level in rotavirus-induced dehydration is higher than that in healthy controls [29]. However, the function of NGAL is not well-known in dengue infection. In addition, NGAL is a biomarker for human acute kidney injury [30]. Although dengue hemorrhagic fever is reported to be a risk factor of acute kidney factor in children [31], the NGAL level does not reveal significant difference between dengue patients and healthy controls in adults. In this study, the dengue patients were enrolled after fever onset. We assumed that most of enrolled patients might be in the acute febrile phase and critical phase of dengue. Relatively low level of all three molecules was observed in dengue patients with leukopenia and thrombocytopenia (Tables 3 and 4). All molecules were significantly correlated with leukocyte and platelet count (Table 5). The highest virus titer is in the febrile phase of dengue (1–3 days post fever onset) and the number of leukocyte and platelet decreases is in critical phase (4–6 days post fever onset) [32]. It might suggest elastase 2, lactoferrin and NGAL play a role in anti-dengue immune responses in febrile phase.

Potts and colleagues report that dengue patients had lower platelet, white blood cell and neutrophil counts [33]. Although we did not count neutrophils in this study, we supposed serum levels of elastase 2, lactoferrin, and NGAL correlated with neutrophil counts in adult dengue patients. A recent study indicated that the concentration of the endothelial cell-related molecule VCAM-1 was negatively correlated with neutrophil count [34]. In Tables 3 and 4, the level of sVCAM-1, IFN-γ and three neutrophils-related molecules showed opposite expression pattern in the leukopenic group and thrombocytopenic group. Thrombospondin-1 which is an inflammatory molecule in activated platelet shows similar expression pattern with elastase 2, lactoferrin, and NGAL in this study [35]. It needs to further investigate whether these molecules involve in the regulation of the interaction between T cells, neutrophils, platelet, and endothelial cells during dengue infection in the future.

Resistin is a kind of adipokine and is involved in various inflammatory processes [36, 37]. Peripheral blood mononuclear cells (PBMCs), macrophages, and bone marrow cells are major sources of resistin in human [38]. Circulating levels of IL-6, IL-10 and IFN-γ associated with the level of resistin in an obese mouse model [39]. Pro-inflammatory cytokines, such as IL-6 and TNF-α induce mRNA expression of resistin in human PBMCs. Resistin is significantly correlated with IL-6 and ICAM-1 in patients with obstructive sleep apnea syndrome [40]. The present study showed that serum resistin level significantly increased in dengue patients. Our results indicated that resistin did not correlate with leukocyte and platelet count although human PBMC and macrophage are reported to secret resistin [38]. Persistent human papillomavirus infection increases the resistin level in plasma of older women [41]. However, the function of resistin in dengue virus and human papillomavirus infection is still unknown. In addition, we observed high expression level of resistin, MIF and sFasL in dengue patients and no significant correlation between these molecules and leukocyte and platelet count. It suggests that serum resistin and sFasL might be potential biomarkers for dengue infection since MIF is correlated with disease severity and clinical outcome in dengue [10].

## Conclusion

We demonstrated that five inflammatory molecules were elevated in the serum from dengue patients. NGAL and resistin were novel dengue-associated molecules while NGAL might associate with anti-dengue immune responses and resistin might be a novel biomarker for dengue infection. Further investigation to determine their roles in dengue infection may contribute to the understanding the regulatory mechanism of anti-dengue immune responses.
